# Effectiveness of prescribed burn health risk communication on community and individual smoke-protective decision making using protective action theories

**DOI:** 10.3389/fpubh.2026.1761725

**Published:** 2026-04-14

**Authors:** Margaux Joe, Ella Anderson, Joanne G. Patterson, Olorunfemi Adetona, Meredith Fritz, Jessica Lemus, Anna Adetona, Elizabeth Babalola, Luke P. Naeher

**Affiliations:** 1 Department of Environmental Health Sciences, College of Public Health, The Ohio State University, Columbus, OH, United States; 2 Department of Health Behavior and Health Promotion, College of Public Health, The Ohio State University, Columbus, OH, United States; 3 Battelle Memorial Institute, Columbus, OH, United States; 4 Department of Environmental Health Science, College of Public Health, University of Georgia, Athens, GA, United States

**Keywords:** community exposure, health education, health risk communication, prescribed burns, protective action decision making, smoke exposure, stakeholder engagement

## Abstract

**Background:**

Prescribed burns are a land management tool with several cultural and ecological benefits. However, they can produce enough smoke to result in adverse health effects increasing the importance of prescribed burn health risk communication. This study evaluated a prescribed burn health risk communication toolkit that is in development.

**Methods:**

Focus groups were conducted with community and institutional stakeholders. The protection motivation theory and precaution adoption process model were then used to interpret focus group responses to understand what factors of prescribed burn health risk communication influence protective action decision-making.

**Results:**

Tailored communications and social context can influence protective action decisions to reduce exposure to prescribed burn smoke. Dissemination of the communication materials from the toolkit may be effective in increasing public health education and communication of prescribed burns and smoke-related health risks, engagement with prescribed burn health risk messages, and smoke-protective decisions. Guidance for involving the community and integrating health risk communication into current practices may increase public engagement with and effectiveness of prescribed burn health risk messaging.

**Conclusion:**

This study provides important implications for public health education and communication about prescribed burns and smoke-related health risks. Although prescribed burns could be considered to have relatively low risk, the resulting smoke is still an environmental hazard of which many communities may not be aware. This lack of knowledge underscores the importance of educational and effective public health messaging so that individuals can make informed protective action decisions.

## 1 Introduction

Prescribed burns (PB) are planned and intentionally set by professionals under appropriate atmospheric conditions for cultural and ecological benefits ranging from increasing wildlife habitat quality, removing invasive species, minimizing pest insect and disease spread, reducing hazardous fuels, and facilitating other land management goals (1–3). While PB benefits are clear, exposure to smoke may lead to adverse health outcomes including respiratory and cardiovascular effects (e.g., coughing, breathing difficulties, stroke, heart failure) and increased emergency room visits and hospital admissions (4). While PBs are planned to minimize smoke exposure, individuals near a PB may be exposed to smoke (5). Protective actions to reduce smoke exposure include limiting time outdoors during a PB, closing windows and doors, using portable air cleaners with high-efficiency air filters (HEPA), and wearing properly fitted N-95 masks if spending long periods of time outdoors (6). Many United States (US) federal, state, and local agencies and organization also use smoke management plans (i.e., framework of procedures and requirements to minimize air quality and public health impacts from smoke produced by PBs) as a tool to inform public health and safety, however, state and local regulations may lead to variations in how these plans address public notification of a PB (4). There is limited research on public health education and communication for PB smoke health risks (7, 8). Previous studies indicate wildland fire smoke-related health risk communication strategies are not well documented, and institutions often face challenges when communicating to the public about smoke, possibly leading to non-existent to ineffective public messaging (8, 9). A systematic review of studies of public health messaging related to smoke events (e.g., wildfire, agricultural burns, prescribed burns, coal fires, structural fires) reported that evidence for effectiveness of communication channels and sources is weak and that research regarding the effectiveness of modern communication channels (i.e., social media posts, text alerts) is even more sparse (9). Communication effectiveness was also identified as a challenge in a study investigating wildland fire smoke communication among land management practitioners (8). The study also found that inconsistent messaging across agencies and internal agency priorities add to the challenges that land managers encounter when communicating about wildland fire smoke (8). These limitations, as well as other factors (e.g., local and social context), could contribute to public misperceptions about PBs, smoke-related health risks, and protective actions (8). Previous wildland fire research reports that local and social contexts, as well as behavior of the individual receiving the information, can impact effectiveness of smoke communication (10–12). However, most of these studies have been conducted in regard to wildfires with only a few including PBs. None of these studies directly address PB health risk communication.

**Figure 1:**
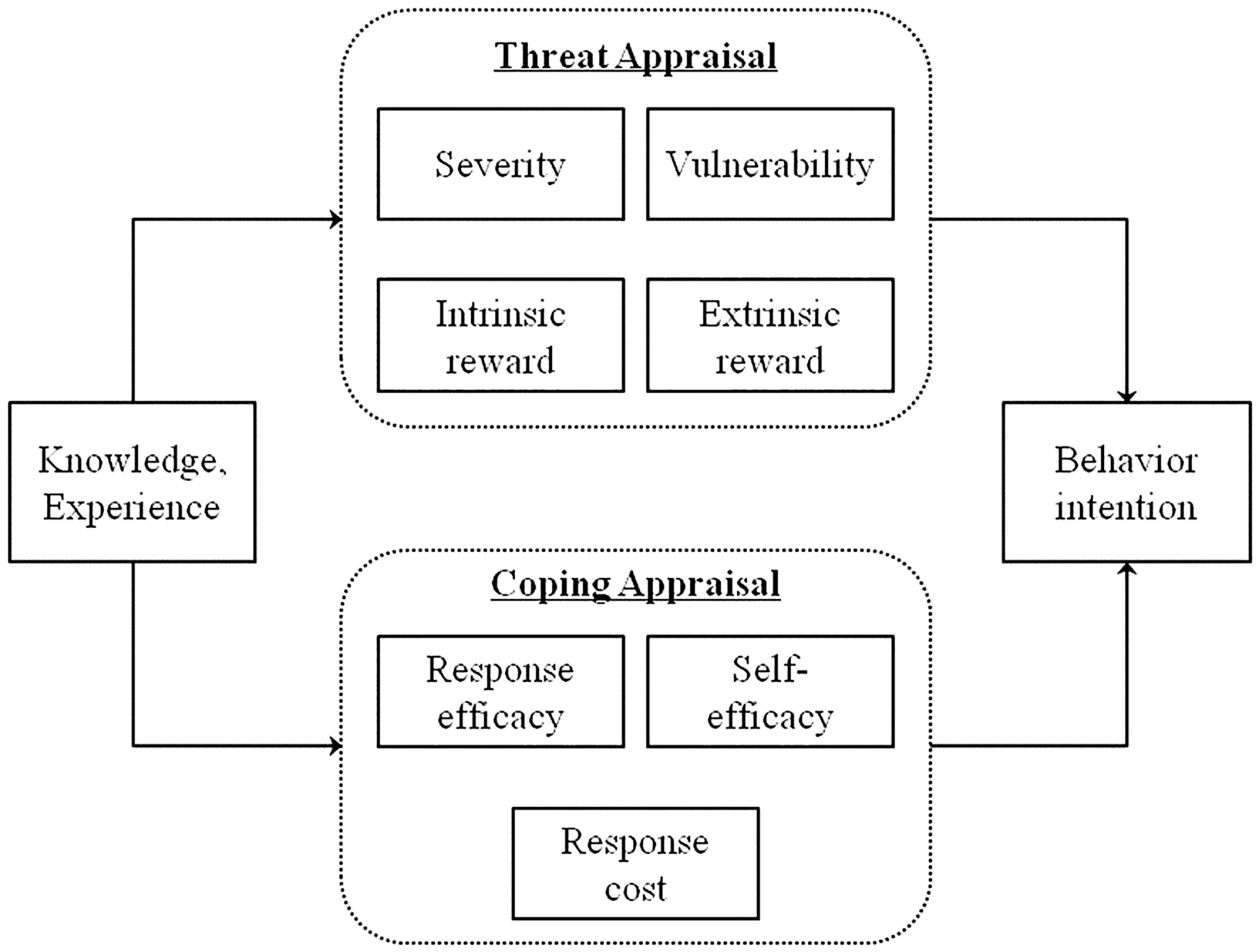
Schematic of the protection motivation theory (56).

The primary objective of this study was to gather feedback about the perceived effectiveness and usefulness of a PB health risk communication toolkit for promoting protective health behavior and decision making. A description of the toolkit and how it was developed is provided in the following section. For this study, we facilitated focus groups (FG) with community stakeholders (i.e., residents living in or near areas where PBs are conducted) and institutional stakeholders (i.e., organizations focused on land management, wildland fire, public health) from across the U. S. As an a posteriori secondary objective, we interpreted the focus group results using two social and behavioral theories to explore how PB health risk communication may influence health risk perceptions and possibly motivate smoke-protective decision making. Published literature indicates that behavioral characteristics can also influence PB health risk perceptions, reception of health risk communication, and adoption of smoke-protective behaviors; however, research in this area is limited (13). Multiple reviews and qualitative cross-sectional studies that have applied social and behavioral theory (e.g., protection motivation theory, Protective Action Decision Model, Theory of Planned Behavior) found that social norms (i.e., informal rules or standards that influence behavior among groups or societies) and perceived self-efficacy are major drivers of adopting smoke-protective behaviors, such as staying indoors or reducing outdoor activity (13–15). This study applies two protective action and social and behvioral theories to understand how behavior may affect use and adoption of PB health risk communication and subsequently motivation to adopt smoke-protective behaviors. The protection motivation theory (PMT) assumes protective action for a health threat is based on the threat’s magnitude, its probability of occurring, and the efficacy of the action to protect against the health threat (16). Therefore, the PMT is postulated to be a function of threat and coping appraisals (16). Threat appraisals represent health risk beliefs encompassing perceived severity, vulnerability, and maladaptive rewards, while coping appraisals represent health promotion beliefs encompassing response efficacy, perceived self-efficacy, and response costs (17). Figure 1 illustrates how knowledge and experience, threat appraisals, and coping appraisals may influence protective behavior intention. While the application of PMT to PBs is sparse, there are studies that have applied PMT to investigate wildfire health risk perceptions and protective motivations (18, 19). Beyond wildland fire, PMT has been widely applied to other areas of public health and health risk communication. Recent studies applying PMT include research focused on flood risk reduction behaviors (20), exercise intent among older adults (21), consumer participation in food safety risk communication (22), and, most notably, risk communication about COVID-19 and COVID-19 vaccinations (23–25).

**Figure 2:**
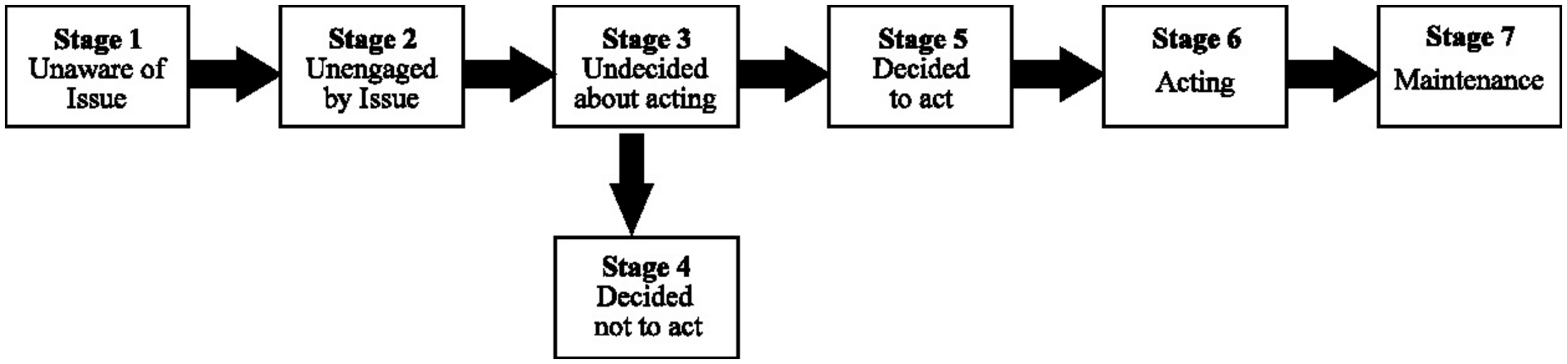
Schematic of the precaution adoption process model (57).

The precaution adoption process model (PAPM) is a stage theory describing how individuals make decisions about adopting protective health behaviors (26). As depicted in Figure 2, the PAPM has seven stages: (1) unaware of the hazard, (2) aware but unengaged, (3) engaged and deciding to act, (4) decided not to act, (5) decided to act but not yet acting, (6) acting, and (7) maintaining the health protective behavior (27). In this model, the hazard does not have to be occurring or imminent, but rather a possible risk that an individual or community should become aware of Weinstein et al. (28). Individuals move through these stages in sequential order, with no minimum time spent in one stage and without skipping a stage but can move backward a stage (27). Application of the PAPM to wildland fire smoke research is limited but has been applied in one study to investigate communication resources about wildfire risks (29). The limited application of PAPM is understandable for emergency disaster hazards (i.e., wildfires) that have high public awareness. PBs, however, are an environmental hazard with little awareness in many areas of the U.S due to its variable use across the country based on the spatial and temporal characteristics of each region’s ecosystem (30, 31). Therefore, using PAPM, which is a model that includes unawareness of the hazard, is applicable in understanding the progression toward smoke-exposure mitigation. While the PAPM has limited application in wildland fire research, it has been applied to study other areas of environmental, occupational, and public health. Studies investigating environmental and occupational health focused on applying the PAPM to study disaster preparedness and education interventions (32, 33), residential radon testing and mitigation (34), and intentions of mine workers to use protective technologies (35). The PAPM has also been applied to investigate breast cancer screening among medically underserved women (36), fall prevention education materials among older adults (37), and vaccination decision making for COVID-19, seasonal flu, and human papillomavirus vaccines (38–40). We used the PAPM to understand the progression for decision-making about smoke protective behaviors and the PMT to understand the factors possibly influencing that progression. The institutional stakeholders were included to understand the decision-making process they undergo when developing and disseminating public communication. The community stakeholders were included to understand how the public receives and interprets the communication and subsequently decides to engage in protective health behaviors. These focus groups are part of a larger multi-stage study with the final objective of developing a resource for PB health risk communication, i.e., the toolkit.

## 2 Methods

The research team conducted three community member (CM) FGs, and four institutional stakeholder (ISH) FGs. Each CM FG had eight participants, for a total of 24 CM participants. The ISH FGs ranged from five to eight participants per group, for a total of 26 ISH participants. ISH participants included PB contractors, university researchers, public health professionals, and land management professionals. The number of FGs and participants for each stakeholder category were determined by, and align with, what has been recommended by best practices published in the literature (41). The difference in numbers per FG between the CMs and ISHs is due to limited resources and availability of the ISHs to participate in the FGs. The Office of Responsible Research Practice at The Ohio State University (Protocol #2022B0055) reviewed and approved this study for human subject research.

### 2.1 Prescribed burn health risk communication toolkit

To address the gaps in PB health risk communication research, we developed a toolkit using health and risk communication theory (i.e., health belief model, risk information seeking and processing model), published literature, and interviews with CMs and ISHs from Ohio, South Carolina, Virginia, and additionally for ISHs from national or federal organizations. CMs are defined as residents living in or near areas where PBs are conducted. ISHs are defined as employees of organizations whose work focuses on land management, wildland fire, and public health. The CM and ISH interviews were conducted between June and August 2023. The Health Belief Model and the Risk Information Seeking and Processing Model were used to develop the interview guides. During the CM interviews (*n* = 18) we asked questions about their PB and smoke-related health risk knowledge, any previous exposure to PBs or PB smoke, previous or planned use of smoke-protective actions, and their information needs for PB health risk communication. During the ISH interviews (*n* = 16), we asked questions about their PB and smoke-related health risk knowledge, their processes for communicating with their audience, existing communication resources they use, their perceived communication barriers, and communication resources they would like to have available. In brief, results from CM interviews indicated they want PB communication materials to include more general PB information (e.g., definition, PB benefits), PB event details (e.g., date, location), smoke-related health risks, vulnerable populations, and recommended protective actions. Results from the ISH interviews suggested they would like resources and guidance for identifying and engaging community partners, as well as integrating health risk communication into existing communication processes. We then triangulated the data from the CM and ISH interviews with best practices from published literature to determine what information to include in the toolkit and how to present that information. The toolkit uses a two-fold strategy that educates PB and public health practitioners about smoke-related health risk communication and includes templates that practitioners can use to communicate with the public about PBs and smoke-related health risks (42). The toolkit includes adaptable materials, such as a list of talking points and pre-designed templates (e.g., burn event flyer and general infographic) with messaging about smoke-related health risks, populations vulnerable to PB smoke exposure and/or smoke-related health risks, and smoke-protective actions. These materials were included in the toolkit based on ISH interview feedback and the information included in the materials was based on CM interview feedback. ISHs also discussed in their interviews wanting tools to help them develop their own messaging, therefore we also included in the toolkit guidance for institutions about developing their own messaging that is effective and engages their communities. This guidance encompasses information about identifying their stakeholders, how to include their community in their communication processes, and a set of checklists to help prepare messages and plan when, where, and how to distribute them.

### 2.2 Stakeholder identification and focus group recruitment

Recruitment (i.e., contact, eligibility screening, and FG scheduling) for all participants was handled by a third-party research logistics company. The geographic study area included Ohio, South Carolina, and Virginia for CMs. In addition to these three states, ISHs were also recruited from Georgia, Kentucky, Kansas, North Carolina, Washington, and national organizations or federal agencies. Recruitment of the ISHs was expanded to the additional five states due to low recruitment rates from the initial three states. Ohio with 1,501–15,000 acres treated annually with PB, Virginia (15,001–50,000 acres), and South Carolina (50,001–200,000 acres) were selected to represent states with low, medium, and high PB activities (43), respectively, and based on the study team’s professional relationships. Within the aforementioned eight states, geographic information system methods were used to identify counties that were in a 5-mile radius of either a state or national park, forest, or refuge. Potential CM and ISH participants were then sampled from counties identified within that radius. We used this approach to ensure that our sample was recruited from areas where PBs could or does take place. All participants were offered a $75 gift card for their participation.

Prior to being scheduled for a FG, all CMs and ISHs participated in an eligibility screening questionnaire with the third-party research company. The screening questionnaires asked questions about age, as well as county and state of residence or occupation (for ISHs) to ensure that all potential participants met the age and location eligibility criteria. Eligible CMs had to be 18 years or older and living in Ohio, South Carolina, or Virginia. The third-party research company leveraged their panel to recruit eligible CMs. This panel is comprised of U.S. residents who have consented to be contacted for participation in ongoing research. Eligible ISHs had to be 18 years or older and working for a relevant organization in the study area (i.e., Georgia, Kansas, Kentucky, North Carolina, Ohio, South Carolina, Virginia, Washington) or a relevant national or federal institution. Organizations were identified using a stakeholder mapping process described in the Supplementary material. Once an organization was identified, members of the study team contacted the organization to determine their interest in participating in the study. The contact information of interested organizations was then given to the third-party research company so that they could officially recruit, screen, and schedule a representative from that organization.

Stakeholder institutions were categorized as traditional (i.e., organizations whose work typically focuses on wildland fire or land management) or non-traditional (i.e., organizations whose work does not typically focus on wildland fire or land management but is adjacent to it or relevant to public health). Examples of traditional institutions included wildland fire management, land management, PB associations (PBAs), and universities conducting wildland fire research. Examples of non-traditional institutions included public health agencies and non-profit or non-governmental organizations (NGOs) (e.g., National Environmental Health Association). We included both types of ISHs to gain comprehensive feedback from professionals in organizations with different primary missions (wildland fire management versus public health) who would still be expected to engage in public health risk communication about PB smoke.

### 2.3 Focus group structure

**Figure 3:**
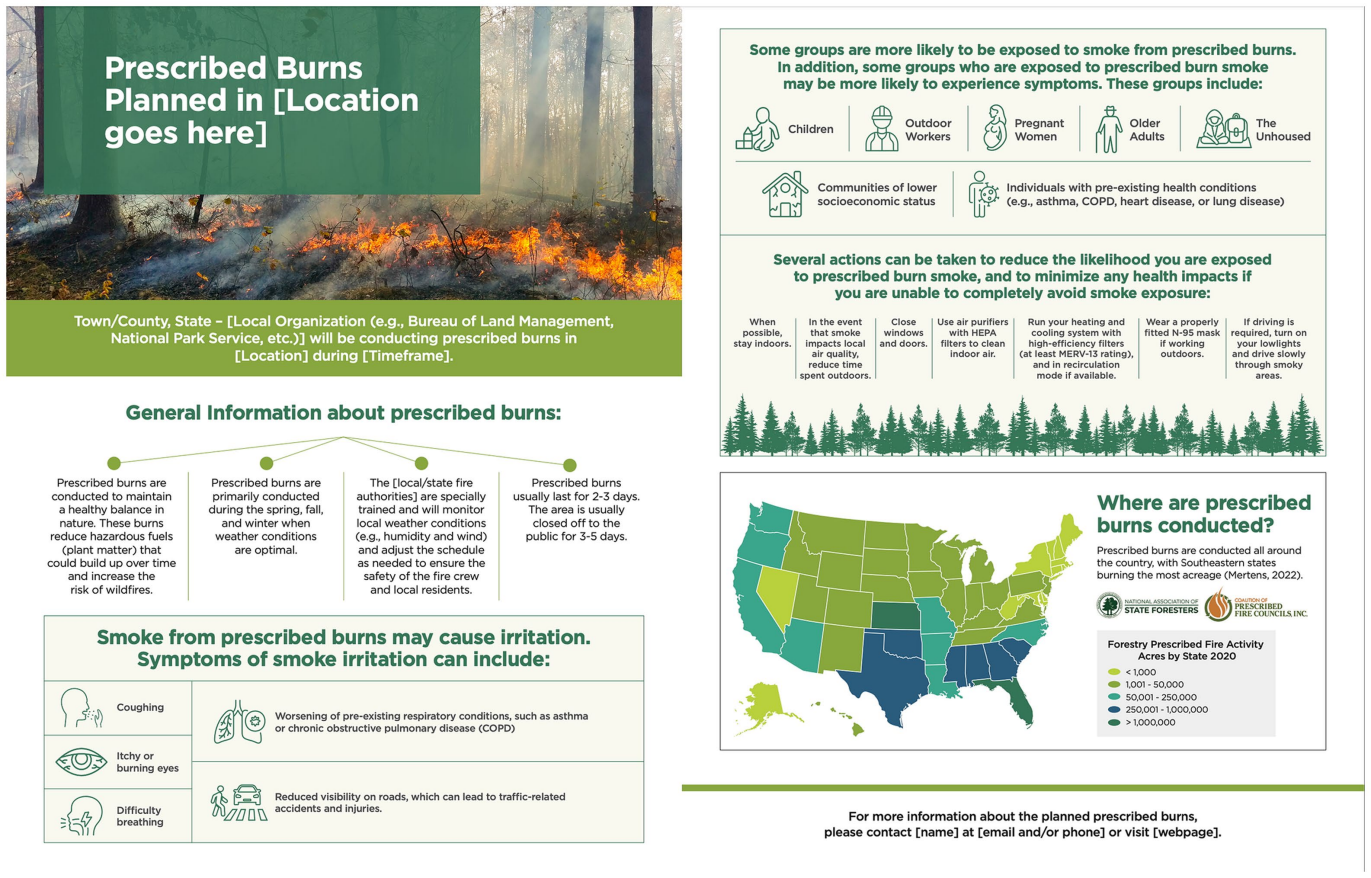
Flyer template shown to the community member focus groups.

**Figure 4:**
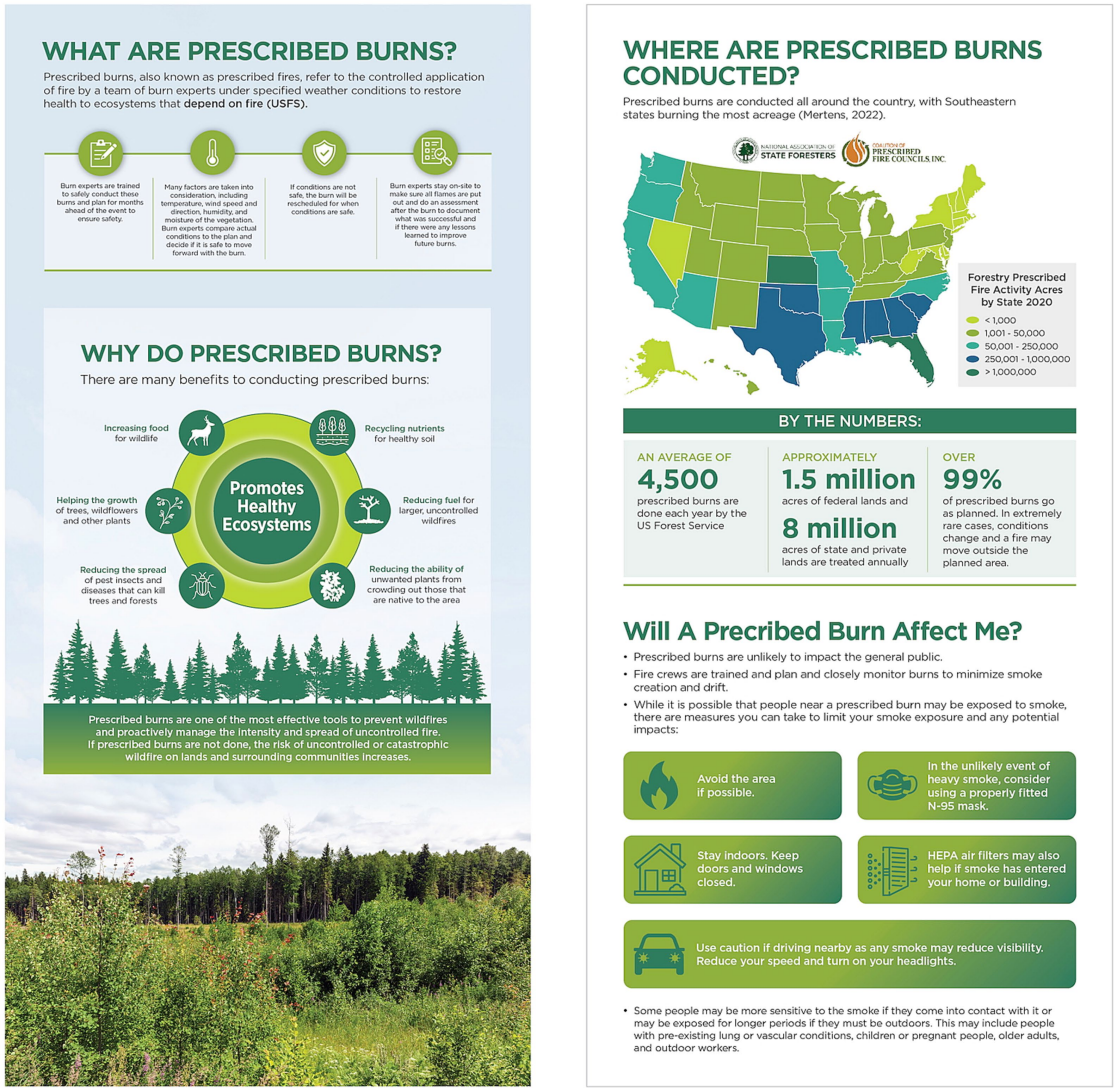
Infographic template shown to community member focus groups.

All participants signed a consent form during scheduling and were asked to reaffirm consent verbally at the start of their FG. FGs were scheduled for 90 min and held over a video conferencing platform hosted by the third-party research company. CM FGs were conducted in June of 2024 and ISH FGs were conducted in February of 2025. Each FG had one facilitator and one notetaker who were both from the study team, as well as a technical advisor from the third-party research company who was present to address any potential technical issues during the session. The number of FGs conducted and number of participants per FG for each stakeholder category are presented in Table 1. Two semi-structured guides were used to facilitate the FGs. The CM guide was designed to gather feedback about a flyer template (Figure 3), infographic (Figure 4), and pre-developed talking points for PBs (Table 2) and smoke-related health risks (Table 3).

**Table 1:** Total number of participants per focus group for each stakeholder category.

Type of stakeholder	Community member stakeholders			Institutional stakeholders			
Focus group #	1	2	3	1	2	3	4
No. of participants	8	8	8	7	5	8	6

**Table 2:** Pre-developed general prescribed burn talking points show to community member focus groups.

Pre-developed general prescribed burn talking points
Fire management teams decide where to conduct a prescribed burn by studying an area ahead of time.
They may choose to conduct prescribed burns in areas they find to be at a higher risk for wildfires compared to other areas.
These areas are at a higher risk because they have a lot of vegetation (e.g., overgrown shrubs, pine needles, grasses, fallen logs, and dead plant materials) that, if ignited, can burn and lead to wildfires.
Fire management teams may also choose to conduct prescribed burns in areas that need regeneration to improve the health of plants, trees, and other vegetation, or to make the land more suitable for wildlife that in turn, support the health of these wildlands.

**Table 3:** Pre-developed smoke-related health risk talking points shown to community member focus groups.

Pre-developed smoke-related health risk talking points
Trained fire management teams conduct prescribed burns under specific weather conditions that allow for more control over the fire’s movement.
Fire management teams can also monitor the fire more closely because it is moving at a slow pace.
These teams are able to limit potential health risks to the public because they can keep the fire away from populated areas or extinguish the fire if needed.
While the amount of smoke generated from prescribed burns is significantly less than the smoke generated from wildfires, prescribed burns do create some smoke.
Smoke exposure can cause smoke irritation, depending on the amount of smoke, how long a person is exposed to it, and how close a person is to the source of the smoke.

**Figure 5:**
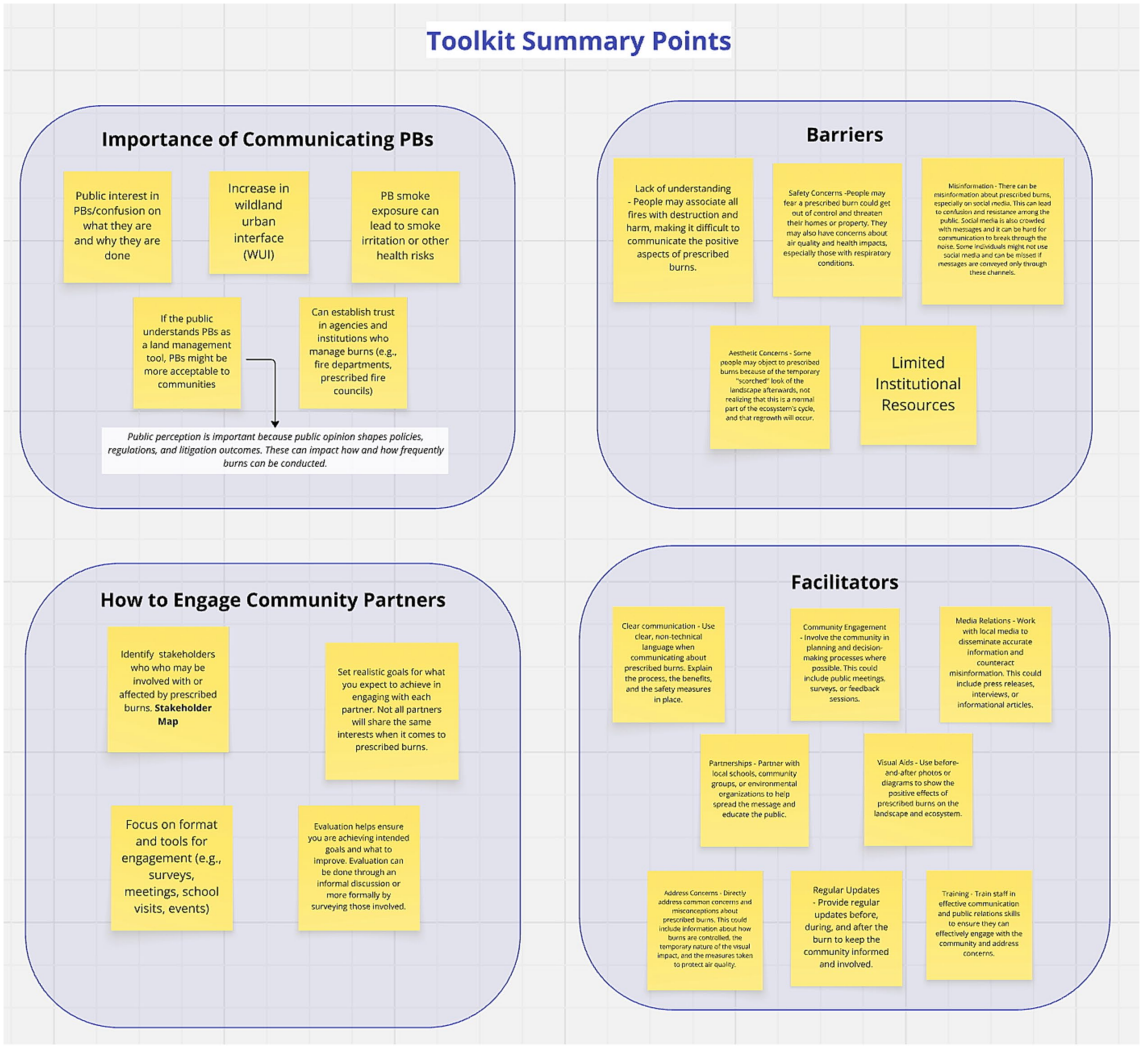
Summary points of communication guidance in toolkit shown to institutional stakeholder focus groups.

**Figure 6:**
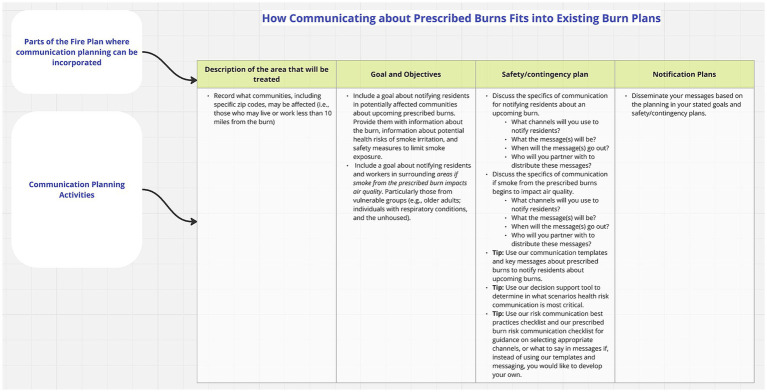
Guidance for integrating health risk communication shown to institutional stakeholder focus groups.

The ISH guide was designed to gather feedback about the communication guidance sections included in the toolkit (Figures 5, 6). Because of the length of the communication guidance sections, we summarized the information into stimuli that could be discussed within the 90-min time range.

### 2.4 Coding and analysis

The codebook for CMs and the codebook for ISHs were developed using their respective FG guides, where the codes in each codebook were determined *a priori* based on the FG guide questions. From the initial reading of the focus group responses, several additional themes emerged from ISH FGs. These emergent themes were added to the ISH codebook and used, in addition to the a priori codes, to code their FG responses. Table 4 presents the emergent theme(s) that corresponds to each ISH FG guide question.

**Table 4:** Institutional stakeholder focus group codes and emergent themes.

Focus group guide section	Focus group guide question (a priori codes)	Emergent theme (a posteriori codes)
Importance of communicating about prescribed burns	How well do these points make the case for why it is important to communicate about prescribed burns? What reasons do you think are missing from this list?	Public Awareness/Acceptance Institutional Trust
Barriers/Facilitators for communicating about prescribed burns	How applicable are these barriers or facilitators to you/your organization? What barriers/facilitators would you add to this list?	Public Awareness/Acceptance Institutional Trust Misinformation Disconnect in Communication
How to engage community partners	Describe your initial thoughts or reactions to these points. Anything you would add to this?	Institutional Trust Social and Cultural Differences
Stakeholder map	Describe your initial thoughts or reactions to this graphic. Are there any stakeholders that are missing from this graphic?	Institutional Trust Social and Cultural Differences Disconnect in Communication
How to fit health risk communication into existing processes	Are the communication planning and activities proposed realistic? Feasible? What could make this more helpful?	Misinformation Disconnect in Communication

Two study team members independently coded the same section of a FG transcript, randomly selected by one of the two study team members, to determine intercoder reliability using Cohen’s kappa of 0.8 or greater, which measures the reliability of two raters to agree on an item’s coding (44). Kappa for the initial independent coding was 0.8, indicating strong agreement. The study team members proceeded to code using a single-coder method. Both researchers used NVivo 14 (45). The codebooks were used to analyze the CM and ISH FG responses for feedback about the toolkit. Although, the primary purpose of the CM and ISH FGs was to obtain feedback about the toolkit, the study team found several connections to the PMT in the CM FG data and associations with the first three stages of the PAPM in the emergent themes from the ISH FG data based on the initial read-through of the coded responses. Therefore, the study team secondarily decided to use the PMT and PAPM to interpret the coded focus group responses to understand factors influencing perceived protective health behavior and decision making. Table 5 presents the PMT constructs used to help interpret the coded responses from the CM FGs. While the PMT constructs were used to interpret the CM FG data, the PAPM was used to connect the responses from the CM FGs to the responses from the ISH FGs.

**Table 5:** Protection motivation theory constructs used to interpret the coded community member focus group responses.

Focus group guide question	PMT construct
What are your impressions of prescribed burn health risks after looking at this flyer?	Perceived severity
What are your perceptions of prescribed burns after looking at this flyer?	Perceived severity Perceived vulnerability
Based on what you have seen so far, what level of risk do you think prescribed burns pose to your health?	Perceived severity Perceived vulnerability
After looking at these materials, what are your perceptions of prescribed burns? Why do you feel that way?	Perceived severity Perceived vulnerability Response efficacy Perceived self-efficacy
How confident would you feel explaining what prescribed burns are to someone else?	Response efficacy
After reading this flyer, what actions would you take?	Perceived self-efficacy
Based on what you have seen, how confident would you feel taking actions to reduce your prescribed burn smoke exposure?	Perceived self-efficacy

## 3 Results

Participant demographics are in the Supplementary material. Table 6 presents exemplar quotes from the CM FGs. Table 7 presents exemplar quotes from the ISH FGs.

**Table 6:** Exemplar quotes by theme from community member focus groups.

PMT construct	Community member focus group quote
Perceived severity	“I think it’s concerning if you have, especially if you have pre-existing conditions or you are older. It would be really worrisome if I had COPD or something like that.” -FG 1
Perceived vulnerability	“My first reaction, because I live with older parents and because I have asthma, is I would start looking really quickly. I’d start right away trying to figure out how bad is it? Do I need to get out of here while this is happening? Do I need to move my family for these few days because of the irritation? What effect is it going to have? The irritation symptoms would alarm me a bit.” -FG 3
Response efficacy	“I feel confident [that I could explain what PBs are to someone else]. I’ve learned something in detail and I like that.” -FG1
	“I’d want to share it. Anybody that I know, I’d want to have the ability of sharing it to the people that I know, work with, family, that kind of stuff. Once I saw it, I’d want to be able to pass it along.” -FG 2
Perceived self-efficacy	“I understand the precautions after seeing the information and I would stay inside with air on, filters, if possible, doors and windows closed.” -FG 2
	“I like that this covers all people. So now we all know what to do and we all can take necessary action if we need to.” -FG 3
Final perceptions and material feedback	“I feel more accepting of them because I understand it’s a preventative measure to prevent larger things such as wildfires. So, I may have more positive view on them now.” -FG1
	“I know more now than I did coming into this.” -FG 2
	“I would say I feel generally positive about them. I do believe that the people who are doing it have been trained and are managing risks as best they can. But I do think it’s important to warn the people who are more vulnerable about things that they might experience and what they could definitely do.” -FG 3

**Table 7:** Exemplar quotes by theme from institutional stakeholder focus groups.

Emergent theme	Institutional stakeholder focus group quote
Public awareness and acceptance	“We are over communicated to and attention is very limited and so how do you deliver a well-researched and well tested message in the limited slice of attention that you might get with people?” -FG 1
	“I think when we are looking at communications design, we know that providing the benefits of prescribed fire helps increase acceptance of prescribed fire. And we also know that when you tell people individual actions that they need to take to protect themselves from poor air quality, when you pair that you have strong risks of reducing prescribed fire acceptance. And so I think the frontier of communications research on this is, how are you delivering messages that both build acceptance of prescribed fire while delivering necessary information for people to protect themselves? And I think that that’s the sticky wicket. There’s no research that helps us on this yet. It has not been done as to how you are pairing these things without degrading the acceptance of prescribed fire. And that’s the health risk communication conundrum.” -FG 2
	“I think what needs to be built out more [in the toolkit] is the ecological context, cultural context, and common language. Some people prefer controlled burns, some people prefer prescribed fire so I think trying to communicate about why we are doing things and what impacts are and how we mitigate those is difficult if you have not already established the ecological context for that and making sure that everyone understands the language you are using. That’s something we have consistently run into issues with here.” -FG 3
Institutional trust	“In the health group again, just sometimes in smaller, more rural communities, having a local health official or a local doctor be involved. Outside of the public health department people might have more trust in someone that they know and is part of their community. Educating directly healthcare providers about prescribed burns might be helpful.” -FG 4
	“One barrier for us as well is we are not the only organization burning in this area. When there is inconsistency across different agencies that are burning, particularly if there’s a crisis like an escaped burn or something that can lead to eroded trust in the public and confusion and rumors and things like that, consistency and communication within an organization but also across different agencies that are burning is really important.” -FG 4
Misinformation	“We’ve tried a lot to incorporate our news media into some of our actual burns and take reporters out on the site to help educate them so we do get a consistent message that this is a land management effort and not a wildfire situation. We try to draw the distinction between the two very precisely so that they understand. One of the biggest problems we have is when the news media reports a wildfire that’s destroying the land and that is not the case. It’s a renewal of the land. It may have destroyed some personal property but it does not necessarily destroy the resource.” -FG 3
Social and cultural belief differences	“But then you go to rural parts of the states, people that have lived here for decades, they are never going to- If they see smoke in the yard, they are never going to call anyone because they just understand that somebody is burning. It’s just it’s been a part of their culture for a very long time here. Luckily, that never went away despite the efforts in the 20s and 30s. So it’s two groups of people, some more rural people that have lived here for decades just understand what it is. But then it’s the folks moving in, mainly from up north that they just do not have the education and it’s not been pointed out to them why we burn, how we burn, where we burn, those types of things. And I’ve encountered folks that, because I’ve burned in urban areas before and they do not understand the level of planning, equipment, training, tools, people, communication, all the things that go into a prescribed burn. Those folks think we are just going out there and throwing a match on the ground and not taking in the consideration transport wind, wind speed, wind direction, relative humidity, temperature, all of these things that go into a very well planned prescribed burn.” -FG 1
	“There is an underlying cultural tension between rural and urban. [PBs are] viewed largely as a rural tool that may have more urban impacts, at least with what we see. We get accused of sending smoke to [urban areas] all the time. So I think there’s some sort of underlying cultural barriers and how you communicate across that urban/rural divide.” -FG 3
Disconnect in communication	“What’s missing is that in much of the east, particularly the southeast, 80 to almost 90% of the land is privately owned and it’s landowners doing the burns, not an agency. And I think a lot of people maybe assume that agencies do a lot of the burning but here, 70% of the land that’s burned every year, maybe more than that is private landowners burning their own property. So that social license to burn and having trust in agencies is a piece of it but a lot of people cannot understand why the farmer down the road is burning his field and why we even let them do that because that’s just a guy dragging a torch. He’s not a trained firefighter so why is he allowed to do that?” -FG 1
	“We’ve done burns inside city limits. We’ve had the fire chief and the rookies on the fire department come and put a drip torch in their hand and they learned about fire. They learned about wildfire because there’s a lot of places within our cities that could burn in a heartbeat if conditions are right and there’s an ignition source. The communication piece, you have got have a consistent, understandable message across multiple platforms so that people can take appropriate action for themselves and their families and their colleagues.” -FG 4

### 3.1 Feedback from community member focus groups

#### 3.1.1 Feedback for health risk information in toolkit materials

FGs liked that the flyer had visuals accompanying the list of smoke-related health risks. They also appreciated that the talking points discussed the PB planning process for minimizing public exposure to smoke. Although they believed the stimuli were informative, the FGs said the talking points could be more concise. Additionally, participants in two FGs wanted more information on the duration of the health effects and when to seek medical assistance.

Some participants described the health risks as concerning, especially for those with pre-existing health conditions. However, most participants felt reassured there are actions to reduce their exposure and potential smoke-related health effects. After reviewing stimuli, most CMs felt they were at low risk of PB smoke affecting their health. Participants in two FGs, however, discussed that their family members with pre-existing health conditions or any individual or group listed as vulnerable on the flyer would have a higher risk of PB smoke exposure affecting their health. None of the participants expressed they were personally vulnerable to the health risks related to PB smoke exposure.

#### 3.1.2 Feedback for protective action information in toolkit materials

All FGs said the protective actions sections on the flyer and infographic were informative and straightforward. They liked the visuals that complemented the protective actions on the infographic (see section “Will a Prescribed Burn Affect Me?” on Figure 4) and felt the flyer would benefit from including those visuals too. They said the phrasing on the flyer and infographic that was used to define and describe PBs reassured them that PBs are not like wildfires such that they can more readily protect themselves from smoke exposure if needed.

After reviewing stimuli, participants in all FGs said the actions they would take to reduce their smoke exposure would be sharing burn event information (e.g., date, location) with their friends and family, staying indoors, using air purifiers, and keeping windows and doors closed. Participants in one FG also said they would look up more information about the burn event, and participants in a separate FG said they would determine if they were in the immediate area and, thus, would be impacted by the smoke. All participants said they felt confident using the protective actions listed on the flyer and infographic.

#### 3.1.3 Final perceptions and overall toolkit material feedback

At the end of each session, participants in all three FGs expressed positive perceptions of PBs, with participants in one FG feeling more accepting of the practice and participants in another FG feeling more knowledgeable. Additionally, most participants believed that PBs have benefits despite the potential smoke-related health risks. Furthermore, all three FGs felt confident they could explain PBs to someone else because the information presented in the stimuli was well-organized and used lay terminology.

Overall, the CMs felt the information presented in the stimuli was informative and increased their awareness of PBs and smoke-related health risks. Of the stimuli presented, all three FGs preferred the infographic because it balanced the PB benefits and the smoke-related health risks. Specifically, all three FGs liked the concise wording and graphics used to visualize the PB benefits because it was simple, yet educational, and kept their attention. While the participants said the flyer was informative, they felt it had too much information. Nonetheless, all three FGs appreciated the organization and the simple terminology in all stimuli.

### 3.2 Feedback from institutional stakeholder focus groups

#### 3.2.1 Overall toolkit feedback

Overall, the FGs thought the toolkit would be useful. Three FGs expressed that the toolkit provided helpful information and would be useful for PB and health risk communication. The remaining FG thought the toolkit would primarily be useful for larger institutions, citing that individual burners and smaller organizations would likely not have the capacity or resources to implement some of the processes outlined in the toolkit. However, those participants felt that it would be a useful educational tool for training new PB practitioners and non-PB practitioners. Because the ISH FGs were focused on the communication guidance portions of the toolkit, we did not show these participants the flyer, infographic, and talking points. However, the ISH participants were provided with the website link to the full toolkit to view on their own if desired. A few participants in two FGs reviewed those communication materials (e.g., flyer and infographic) and said they would be useful because it is often challenging for practitioners to select the most effective wording and content.

Participants in all four FGs agreed with the list of reasons for why PB communication is important. They noted this stimulus was missing information about the PB planning process, examples of who conducts PBs, protective actions for smoke exposure, and guidance about identifying populations at risk of smoke exposure. While details of these topics were not included in the summarized stimulus, they are included in the full toolkit. They also suggested the list include cultural reasons for PBs, additional explanation about the wildland-urban interface (WUI), and explanation about smoke management plans. The FGs also agreed with the list of barriers to and facilitators of PB and health risk communication. However, one group noted these lists primarily apply to agencies and organizations and not necessarily individual burners (e.g., private landowners, certified burn managers). They explained that individual burners often lack resources for public communication, and some burners may not be fully aware of the importance of communicating about smoke-related health risks. Additional barriers they recommended for consideration include technological literacy, language barriers, consistent inter-agency messaging, identifying appropriate communication methods and channels, and navigating the surplus of information communicated to the public. The FGs also suggested including partnering with trusted community organizations or leaders to communicate PB messages as facilitators.

FGs felt the steps for engaging community partners outlined in the stimuli are part of a general method that practitioners typically follow. While they agreed, they noted these steps should include identifying the partner’s interests and priorities and knowing the community’s history. One FG also mentioned when going through this process, it is important to remember that several communities are heterogenous and contain multiple sub-communities. Therefore, this process may need to be conducted multiple times for one area.

Most participants said the stakeholder map, which is the same map we used during our stakeholder mapping process (Supplementary Figure S1), was comprehensive, but a few thought it was overwhelming. Across FGs, participants suggested the following stakeholders be added: community organizations (e.g., public libraries, churches), individual burners, PBAs, and healthcare professionals. Two FGs noted it would be beneficial to include an explanation on how to use the map, as well as examples of the stakeholders. For existing communication processes, participants in all four FGs said the steps presented are typically what they include in their PB plans for general communication. One FG mentioned, however, this process is more applicable to a single burn event but does not work well for a burn season across an entire area. Another FG mentioned there would need to be more detail and planning for larger PBs than what the stimulus presents. Neither FG provided further details to improve the process. Participants across FGs said this process would be helpful for newer practitioners or as a checklist for experienced practitioners to ensure they are not missing steps.

During each session, we demonstrated how to use our decision tool prototype. The purpose of this version of the tool was to guide practitioners on when to disseminate health risk communications based on real-time monitoring of the air quality index. Participants noted it would be beneficial to include instructions for whom the tool is intended and how to use it, as well as the recommended communication tools. The FGs had mixed comments about the usefulness of this tool. Some participants felt it was only useful for larger organizations.

#### 3.2.2 Emergent theme: public awareness and institutional trust

Additional themes emerged in the FGs including institutional trust, public awareness, social and cultural differences, misinformation, and communication disconnection. Participants across FGs emphasized that public awareness and institutional trust are important factors influencing PB and health risk communication. Participants across FGs said the public is often confused about the difference between PB and wildfire, as well as what is the WUI. Two FGs discussed that as suburban areas grow closer to rural areas, newer residents tend to confuse PBs with other types of burns (e.g., agricultural burns) or not understand the importance of PBs, so they are unaware of their benefits and the PB planning process. Another FG noted the importance of increasing public awareness of smoke-related health risks and the PB planning process so the public can make informed protective action decisions since they can potentially experience smoke-related health effects. All FGs discussed that PB awareness is often tied to acceptance of PB use. One FG expressed that little communication reaches the public, therefore, when some residents see the burn, they associate it with the wildfires seen in the news leading them to disapprove of PB use. All four FGs discussed how additional public education and improving institutional trust may relieve confusion and increase general PB awareness, as well as awareness of smoke-related health risks and protective actions. They said communicating about the planning process for smoke management and PB events may reduce the confusion between PBs and wildfires, as well as community member concerns about smoke in the air. Two FGs said that institutional trust also plays a role in PB awareness and acceptance. While the FGs were not specific about what affects institutional trust, they did allude to inconsistent communication between managing agencies as a contributor to it. They described that an increase in transparency between agencies managing the PB and the public can improve the overall social acceptance and institutional trust which may lead to increased PB and smoke-related health risks awareness and normalization of PB use.

#### 3.2.3 Emergent theme: public engagement and decision-making

Participants indicated that misinformation and social and cultural differences influenced both PB perceptions and how the public engages with PB use and communications. Geographical differences, especially rural versus urban, was the primary factor discussed across FGs. Participants indicated that individuals more familiar with PBs sometimes overlook PB communications. Three FGs discussed this is often the case in areas with a strong burning culture such as rural communities and Native American communities. They explained it is not because these communities think general PB communications are unimportant, but rather they have a long history of PB experience, therefore, some of the messages feel redundant. They noted this is a barrier that could also affect smoke-related health risk communications. All FGs discussed the differences in communication channels between rural and urban areas. Participants highlighted that in many rural communities the only way to communicate with nearby residents is via door-to-door knocking, landline phone calls, or posted flyers in frequented local spaces (e.g., post office) since many residents may not use social media or lack internet access.

Disconnect in communication (i.e., gap in understanding between groups where messages are not being received or interpreted as intended) and misinformation are other themes that arose in FG sessions. All FGs discussed how wildfire news has contributed to public misunderstanding that all fire is bad. They explained that because of this many individuals see PB smoke and think something is burning without reason when it is a planned burn event with environmental goals and strategies to manage public smoke exposure. Participants suggested that much of this misunderstanding could be related to a breakdown in connection between institutions and the public, which has led to a gap between the PB messages communicated and what the public understands from the messages. They indicated that inconsistent messaging between organizations and across communication platforms makes it unclear why PBs are being conducted, what are the health risks, who may be impacted, and the decisions individuals can make to protect against smoke exposure. During these discussions, participants suggested that the guidance and templates provided in the toolkit, such as the stakeholder map and flyer template, could help institutions tackle the challenges highlighted in FG sessions.

### 3.3 Application of protection motivation theory and precaution adoption process model

**Figure 7:**
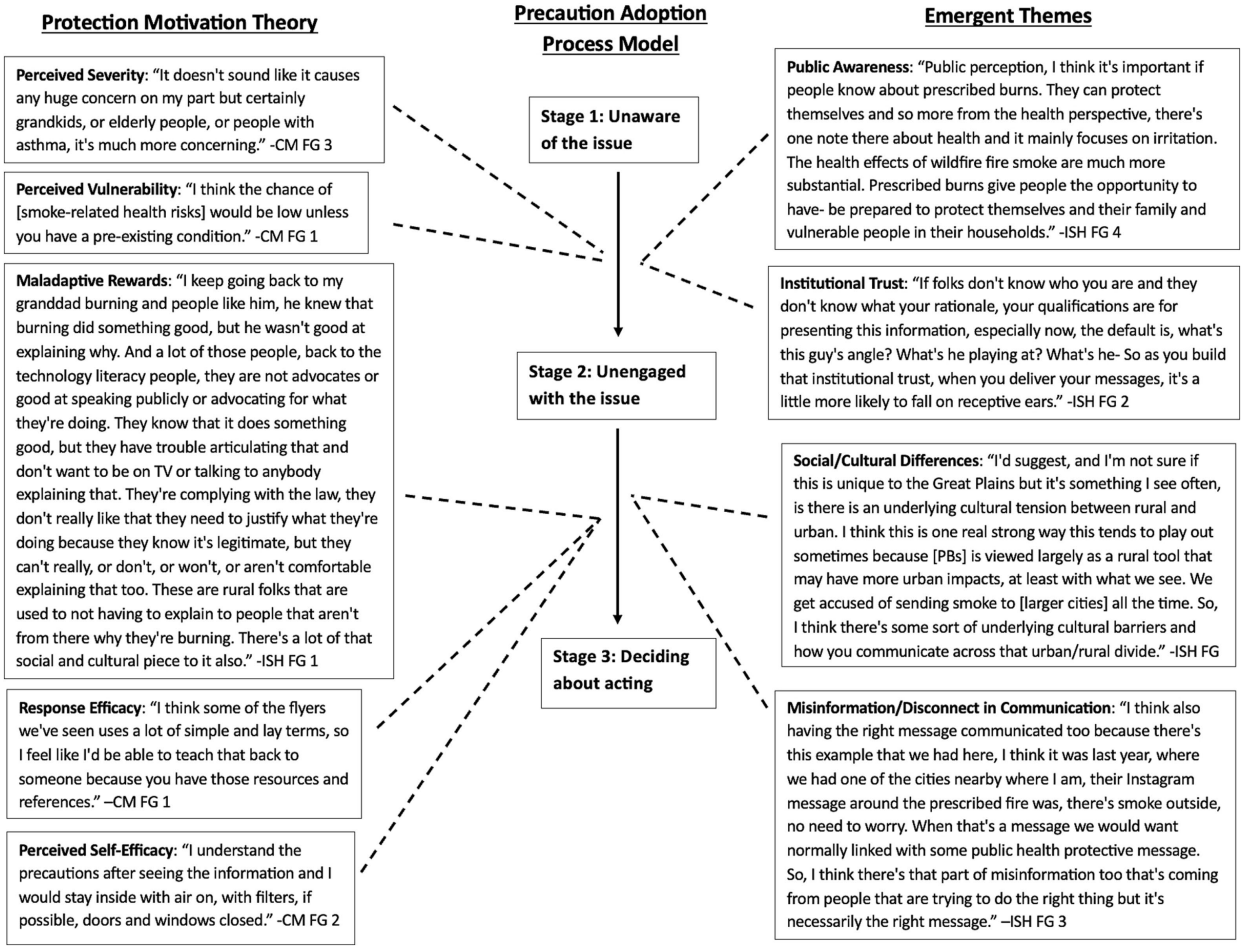
Interaction between PMT and PAPM for health behavior change and smoke-protective decision making based on focus group responses. Solid arrows indicate movement between the PAPM stages and dashed lines suggest where the PMT constructs and emergent themes may influence that movement based on focus group responses.

Interpretation of our results a posteriori using the PMT and PAPM indicates that the PMT constructs of perceived severity, perceived vulnerability, maladaptive rewards, response efficacy, and perceived self-efficacy, as well as emergent themes from the ISH FGs, are factors that may encourage movement between the first three stages of the PAPM (i.e., awareness, engagement, and decision-making). Figure 7 depicts the interaction of the two models in this study as illustrated by FG responses. Across all three CM FGs, participants said their awareness and knowledge of PBs, smoke-related health risks, vulnerable populations, and protective actions increased after reviewing the toolkit materials (i.e., flyer, infographic, talking points). CM participants in all three FGs also indicated that the toolkit materials were engaging and educational such that they could explain the information on the materials to others. These results suggest that perceptions of severity and vulnerability, constructs of the PMT, may motivate movement between Stage 1 and Stage 2 of the PAPM. ISH FG feedback also suggests that institutional trust and public awareness of PBs are additional factors that may encourage movement between Stage 1 and Stage 2.

CM FG results suggest that gaining knowledge of smoke protective actions may encourage increased perceptions of response efficacy and self-efficacy, thereby motivating movement between PAPM Stage 2 and Stage 3. However, ISH FG results indicate that socio-cultural differences, misinformation or disconnect in communication between institutions and communities, and maladaptive rewards (e.g., never having experienced smoke-related health impacts) can impact the effectiveness of PB communication; subsequently affecting movement between Stage 2 and Stage 3, and ultimately deciding whether to take smoke protective action.

## 4 Discussion

We developed a PB health risk communication toolkit with communication guidance and pre-developed templates based on best practices from published literature and stakeholder interviews. This study evaluated the perceived effectiveness and usefulness of the toolkit via stakeholder FGs and used the PMT and PAPM to interpret coded responses to understand how the toolkit may influence perceived smoke protective behaviors and related decision-making. Although the PAPM is a seven-stage model, this study focuses on the first three stages of being unaware about PBs, becoming aware but not engaged, and deciding to act. Our findings suggest that the PMT constructs, as well as the emergent themes from the ISH FGs, may motivate individuals to move between the three PAPM stages. More detailed interpretations of how the PMT and PAPM were used to understand the perceived effectiveness and usefulness of the toolkit are below.

### 4.1 PAPM Stage 1: becoming aware of PBs and smoke-related health risks

PB health risk communication can impact PMT threat appraisals which may, along with other social factors, subsequently influence movement through the PAPM stages. As depicted in Figure 7 and as illustrated by the quotes in the figure, we infer that perceptions of severity and vulnerability (from the PMT), as well as public awareness and institutional trust (emergent themes) may increase community awareness of PBs and smoke-related health risks. The CM FGs suggest that PB health risk communication materials can help individuals identify if they are vulnerable or susceptible to PB smoke exposure and related health risks, and if so the potential severity of those health risks. Responses from the CM and ISH FGs suggest institutional trust and communication materials containing content about the PB planning process and benefits may influence movement from Stage 1 to Stage 2. The exemplar quotes in Figure 7 illustrate that institutional trust influences how receptive the public is of PB health risk communication and how these messages can increase public awareness and perception of smoke-related health risks and protective actions.

Although they lived in areas where PBs were most likely to be conducted, many of the CMs were unaware of PBs or understood very little about the practice at the start of the FGs. The ISHs affirmed that PB awareness in many communities in which they work and other areas across the US was low. While the CMs did not specify why they were unaware of PBs, ISHs perceived the lack of PB awareness and acceptance likely results from institutional distrust and disconnect in communication, which may be affected by misinformation, social and cultural differences, and inconsistent communications. Although ISHs did not always provide detailed responses about what affects PB awareness and institutional trust, we deduce why these gaps may exist based on the results from previous research. A qualitative study using focus groups with residents of wildfire prone areas found that institutional trust is often associated with open and frequent communication while distrust is associated with lack of communication (46). The ISHs did not comment on their communication frequency but did discuss that inconsistent communication among and between institutions can affect if and how the public receives PB messages. Similar findings were reported from research conducted in Australia that suggests inconsistent bushfire communication approaches can undermine confidence in environmental and health authorities (11). This is consistent with other literature conducted in the US that found institutional distrust and inconsistent communication influences PB health risk perceptions, access to information, and adoption of protective health behaviors (10, 12). Standardization of wildland fire communication protocols across institutions can potentially resolve the reported communication inconsistencies and subsequently increase institutional trust. However, as suggested by our participants, standardizing protocols across multiple fields and organizations can be challenging. The communication guidance and pre-designed materials, though, can potentially inform how those barriers can be overcome since they were developed from the input of multiple diverse stakeholders.

PB research also found that individuals familiar with PBs, especially its benefits, and those who trust institutions tend to have more positive perceptions of PBs (31). Similar results were found in this study, even though many CM participants were not familiar with PBs prior to the FGs. As illustrated with the exemplar quotes in Figure 7, the CMs perceived their personal vulnerability and severity to be low. However, after reviewing the communication material stimuli (Tables 2, 3; Figures 3, 4), CM participants expressed an increase in their awareness of PBs, smoke-related health risks, vulnerable populations, and smoke-protective actions. This suggests that the communication materials in the toolkit may be effective for increasing awareness about PB and smoke-health risks. These findings align with research that reports exposure to communication resources about wildfire smoke risks can motivate movement between PAPM stages of issue awareness, engagement with communication, and taking protective action (29). However, unlike other wildfire research that reports wildfire knowledge was a positive predictor of smoke-protective behaviors through increased perceptions of PMT threat and coping appraisals (18), we did not see any thematic associations in the FG data between PB knowledge and perceived smoke protective action. This difference in our findings and those of published wildfire research is likely a result of the low awareness and relatively low risk of PBs compared to wildfires in that gaining awareness can increase perceived vulnerability, but perceived severity is impacted minimally due to the relatively low risk nature of PBs. Personal experience with PBs and sensitivity from pre-existing health conditions may also be influential predictors of adopting protective behaviors to reduce PB smoke exposure (11). We cannot confirm this, though, since we did not ask about susceptibility factors, wildfires or the comparison of wildfires to PBs during the FGs. Nevertheless, we can deduce from the results that PB health risk communication toolkit materials may increase awareness and engagement.

### 4.2 PAPM Stage 2: engaging with PB and smoke-related health risk communication

As depicted in Figure 7, maladaptive rewards (PMT threat appraisal) and local and social contexts may influence reception of and engagement with PB health risk communication (i.e., movement from Stage 2 to Stage 3). ISHs highlighted the importance of considering the community’s history, interests, and priorities (i.e., local and social contexts) when engaging community partners and communicating about PBs and smoke-related health risks. This finding is consistent with previous research that reports local, individual, and household contexts (e.g., institutional trust, community-specific risks and history, lived experience, personal health) can inform PB risk perceptions and related protective action decisions (10, 11). The ISHs discussed that these contexts can affect public engagement with PB communication and protective action decision-making in addition to PB awareness. This aligns with other published work investigating bushfires in Australia which found that personal bushfire experience, sensitivity from pre-existing health conditions, and community-specific risks were predictors of awareness and adoption of smoke-protective behaviors (11). These patterns are likely related to a variety of factors, but most probably to social norms and maladaptive reward systems. For example, individuals who have not experienced PB smoke or adverse smoke-related health effects are likely to believe they will not experience these risks in the future. Additionally, social norms can drive attitudes and behaviors toward wildland fire and smoke-related health risk communication (13). For example, in areas where PBs are common, residents may not view smoke as a health risk but rather as a temporary, acceptable part of land management and therefore may lead to lower compliance with health guidance. This is a situation that our ISHs discussed in their FGs especially among rural residents. ISHs said many rural communities have a long history of PB use, however, not all communities are aware of the smoke-related health risks, or do not perceive the benefits of protective action decision-making to be pertinent (e.g., maladaptive rewards). Examples of this sentiment are illustrated with the exemplar quotes in Figure 7. They explained health risk communication is challenging because local communities may perceive the severity of smoke-related health effects and their personal vulnerability to be low due to their history of PB use, familiarity with smoke in the air, and perceived minimal health impacts from smoke exposure. These results are similar to research from Flint Hills, KS that found rural residents perceived the severity of smoke-related health risks to be low compared to their perceptions of other risks (e.g., liability risk) (12). Another reason health communication can be challenging is that rural areas have limited communication channels, so residents may have limited engagement with PB health risk communication impacting perceived maladaptive rewards and protective action response efficacy and self-efficacy. One way to circumvent these challenges is to incorporate processes for health risk communication into general PB communication procedures, which is a feature provided in the toolkit (Figure 6). Institutions can potentially seamlessly adopt the steps for health risk communication recommended in the toolkit (Figure 6) since the ISHs said the communication processes outlined in the stimulus are what they use for general PB communications.

### 4.3 PAPM Stage 3: deciding to take protective action

Findings support that PB messages influence decisions to take smoke-protective action (Stage 3 of PAPM). Specifically, PMT coping appraisals (i.e., response efficacy and perceived self-efficacy) and addressing potential disconnect in communication may motivate individuals to make smoke-protective decisions (Figure 7). In particular, CMs discussed that using lay terms and making clear what actions can reduce smoke exposure increases their perceptions for the efficacy of those actions and their ability to perform those actions. CMs liked the time and place-based materials provided in the toolkit and appreciated the direct and informative content because it gave them a clear understanding of what is happening and why, if they are vulnerable, and why they may need to protect themselves against smoke exposure. This finding suggests that including this information in communication materials can increase perceived self-efficacy and response efficacy, subsequently motivating protective action decisions. Our findings are supported by other wildland fire smoke literature that reports that clear outlining of smoke-related health risks and protective actions in PB communication materials and the tailoring of these communications to the location and burn event can encourage mitigative decision-making among communities (9, 11). Additionally, our findings align with literature that suggests that using methods for bidirectional communication (e.g., public meetings) can help communities take appropriate protective action decisions, especially communities at higher risk of smoke exposure and related health effects (9, 11). In fact, as described with the response efficacy and perceived self-efficacy exemplar quotes in Figure 7, many CMs indicated that after reviewing stimuli, they felt confident they would make protective action decisions if PB smoke were to impact them, even citing the actions they would take. Previous research reports that utilizing the aforementioned forms of communication can heighten perceived severity of smoke-related health effects, personal and community vulnerability, and protective action response efficacy and self-efficacy (11). The disconnect in communication exemplar quote in Figure 7 has a similar sentiment and explains that it is important to use the right messaging so communities are able to identify and recognize their vulnerability and potential protective actions they can use to reduce their smoke exposure. The toolkit provides these forms of communication in a customizable flyer and talking points that practitioners can tailor to their communities when disseminating PB health risk communications. The toolkit also highlights the importance of community engagement during the planning and decision-making process and provides examples of bidirectional communication (e.g., public meetings, feedback forms or sessions). However, the toolkit does not provide procedural details since it will highly depend on the institution’s resource capacity, as noted in the ISH FGs, and local and social context of the community. Our stakeholder map, though, may be beneficial in those situations since it can be used to identify potential collaborators.

### 4.4 Future directions, applicability, and limitations

The findings of this study provide helpful insights into the factors influencing health risk perceptions, as well as reception and interpretation of PB health risk communication. However, there are limitations. Selection bias may be present across both the CM and ISH FGs. Although we sought a relatively balanced mix of traditional and non-traditional ISHs, we were unable to recruit some institutions (e.g., community organizations for at-risk groups, tribal networks) due to lack of response or declines to participate. Since the FGs were conducted online and a convenience sample was used to recruit CM participants from the third-party research company’s panel, any person not part of the panel and those lacking digital literacy or internet access may not have enrolled in the study. Additionally, since the FGs were designed to elicit feedback about the toolkit, the PMT and PAPM were only used to interpret our results a posteriori and did not use these theories to develop the FG guides or codebooks. Although this limited our ability to analyze the results, we believe we were still able to draw important implications for PB health risk communication since many of the themes from the FG results aligned with constructs from these theories. Further, this study primarily focused on perceived effectiveness. Therefore, we were only able to use the first three stages of the PAPM to interpret our results. Future research should be extended to include the entire PAPM to understand the full decision-making progression from becoming aware of PB health risks to adoption of smoke-protective behaviors and finally to maintaining those behaviors. Future research should also include a more inclusive sampling method than a convenience sample to reduce selection bias and increase sample diversity. We also acknowledge that the qualitative nature of this study does limit the ability to quantify and assess the relative strength of our results. However, we believe these findings are still relevant because they provide contextual insight that can assist in future quantitative studies to evaluate this toolkit and other future research related to PBs and smoke-related health risks.

The CMs and ISHs appreciated several aspects of the toolkit but also provided feedback for improvements. While this is not a limitation of the study, this feedback does provide insightful improvements and future directions for the PB health risk communication toolkit. The CMs held positive perceptions for the flyer, infographic, and talking points, but also felt the wording could be more concise and include more visuals. The ISHs’ main feedback was to make the toolkit more inclusive of smaller institutions and individual burners, as well as for entire burn seasons. While they did not provide details for these improvements, they alluded to including guidance on how to use limited resources and communication procedures for a burn season. Feedback for the decision tool included instructions for who should use it, as well as when and how to use it. Currently, the prototype is for real-time communication dissemination. However, based on feedback, the decision tool could be re-designed for pre-season or pre-burn communications. All feedback will be considered and implemented before sharing the toolkit with practitioners. Updates to the materials and communication guidance will be made available on the toolkit website as revisions are completed.

Although the toolkit was designed with a focus on PBs and smoke-related health risks, findings from this FG study can be more broadly applied to public health and health risk communication. One of the major topics of discussion in this study was the challenge of inconsistent messaging across institutions. Our findings suggest that inter-agency collaborations and using simple, consistent, and direct communication, along with visual explanations where appropriate, can increase engagement with health and risk communication. Although these insights were found while conducting wildland fire research, they can easily be translated to other areas of public health such as messaging for COVID-19 and disaster response and management (47–49). Another major topic of discussion in this study is institutional trust and community engagement. Our findings suggest that engaging with community members, either through education or involvement in program processes, can increase institutional trust and subsequently engagement with communication materials. These findings can also be applied to other areas of public health and risk communication since they are not rooted in fire science research. They are in alignment with those of extensive research, both quantitative and qualitative, investigating institutional trust and natural or technological disasters (e.g., hurricanes and nuclear incidents), as well as growing research on COVID-19 and its impact on institutional trust (50–55). This suggests similarities of influences on public health risk communication between well researched accidents or disasters and events that do not have as much evidence relative to effective risk communication.

## 5 Conclusion

This study provides important implications for public health education and communication of PBs and smoke-related health risks. PB smoke is an environmental hazard with little to no awareness among many communities. As PB use increases, more individuals may be impacted by smoke potentially leading to adverse health effects and increasing the importance of PB health risk communication. It is important that PB health risk communications are informative and effective so that individuals can make informed decisions about protective actions. We conducted this study to evaluate the perceived effectiveness of our PB health risk communication toolkit. Results indicate that our communication materials are effective in raising PB awareness and communicating about PBs, smoke-related health risks, vulnerable populations, and protective actions. The results further indicate that our communication guidance may help some institutions increase their engagement with community partners and PB health risk communications.

## Data Availability

The raw data supporting the conclusions of this article will be made available by the authors, without undue reservation.
