# Supplementary material for: Effectiveness of prescribed burn health risk communication on community and individual smoke-protective decision making using protective action theories
Source: Front Public Health. 2026 Apr 14;14:1761725. doi: 10.3389/fpubh.2026.1761725 (PMC13121289; doi:10.3389/fpubh.2026.1761725)
Supplement: Supplementary file 1 [file Supplementary_file_1.docx]

Supplementary Material

# Supplementary Methods: Stakeholder Mapping Process

We used a stakeholder mapping process to identify relevant stakeholder institutions. This process is used to identify, categorize, and prioritize key institutions or individuals with an interest in the successful implementation of a project – whether contributing to a project directly or potentially affected by the outcomes (**Figure S1**). It allows for a more comprehensive understanding of the viewpoints and motivations of key stakeholders, setting the foundation for stakeholder-driven research, and for more successful and sustainable communication and engagement efforts. Stakeholder mapping was initially conducted by the study team, leveraging professional knowledge and experience, as well as professional networks. It was also supplemented by additional research via Google, for agencies and organizations working in prescribed burns, health communication, or public health to ensure a comprehensive representation of perspectives. This was also coupled with review from an Expert Advisory Panel that provided input at several points in the project, including potential organizations and agencies to contact. The advisory panel consists of four members who have experience with prescribed burns and health risk communication. Any additional stakeholders identified throughout the project lifecycle were incorporated accordingly.


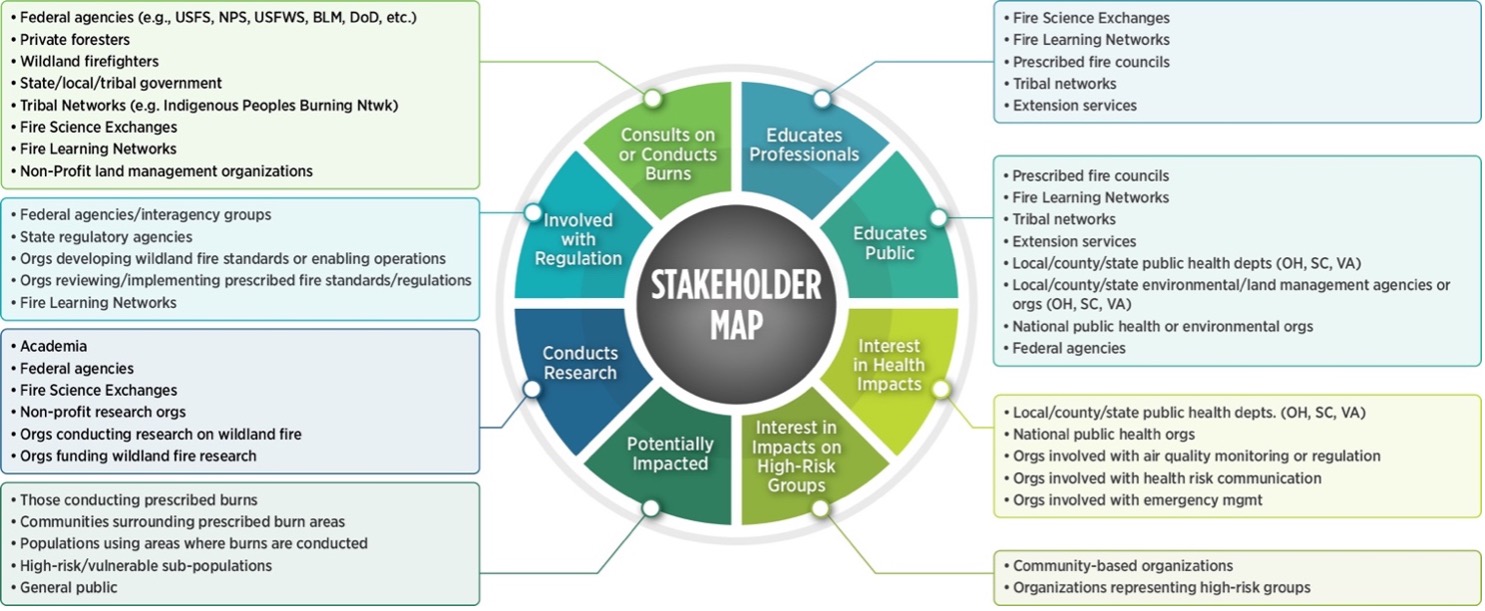


Figure S1. Stakeholder map identifying the traditional and non-traditional stakeholder institutions involved or impacted by prescribed burn communication and health risk

Employees occupying relevant positions at institutional stakeholders identified from the stakeholder mapping were sent an introductory email that included information about the study, how to contact the study team with questions, a link to the study website for additional information, and a notice that they would be contacted by the vendor with additional details regarding participation in a focus group. Interested institutional stakeholders (i.e., stakeholder participants) were then screened via phone or email to determine eligibility for participation. Those who indicated they were not interested in participating did not receive additional communications. Employees at institutional stakeholders referred to the study team by a focus group participant or by an institutional stakeholder who was contacted but had not participated in focus group also underwent the process described above.

# Supplementary Tables

Table S1. Community member focus group demographics

|  | Focus Group 1 | Focus Group 2 | Focus Group 3 |
| --- | --- | --- | --- |
| **State** |  | | |
| Ohio | 2 | 2 | 3 |
| South Carolina | 2 | 4 | 3 |
| Virginia | 4 | 2 | 2 |
| **Gender** |  | | |
| Male | 3 | 2 | 3 |
| Female | 5 | 6 | 5 |
| **Race/Ethnicity** |  | | |
| White/Caucasian | 5 | 6 | 6 |
| Other | 3 | 2 | 2 |
| **Age (Years)** |  | | |
| 18-38 | 2 | 2 | 1 |
| 39-58 | 4 | 4 | 3 |
| 59+ | 2 | 2 | 4 |
| **PB Familiarity** | | | |
| Not at all familiar | 4 | 4 | 2 |
| Somewhat familiar | 4 | 2 | 6 |
| Very Familiar | 0 | 2 | 0 |

Table S2. Institutional stakeholder focus group demographics

|  | Focus Group 1 | Focus Group 2 | Focus Group 3 | Focus Group 4 |
| --- | --- | --- | --- | --- |
| **State** |  | | |  |
| Georgia | -- | -- | 1 | 1 |
| Kansas | -- | -- | 2 | 1 |
| Ohio | 1 | 2 | 1 | 1 |
| South Carolina | 2 | -- | 3 | -- |
| Virginia | 2 | -- | -- | -- |
| Washington | -- | 1 | -- | 3 |
| National/Federal | 2 | 2 | 1 |  |
| **Gender** |  | | |  |
| Male | 5 | 2 | 6 | 1 |
| Female | 2 | 3 | 2 | 5 |
| **Race/Ethnicity** |  | | |  |
| White/Caucasian | 6 | 3 | 8 | 6 |
| Other | 1 | 2 | -- | -- |
| **Institution Type** |  | | | |
| Traditional | 5 | 3 | 3 | 1 |
| Non-traditional | 2 | 2 | 5 | 5 |
